# The relationship between adult phenylketonuria and the cardiovascular system - insights into mechanisms and risks

**DOI:** 10.1186/s13023-025-03686-4

**Published:** 2025-04-02

**Authors:** Yann Dos Santos, Friedrich Trefz, Maria Giżewska, Annemiek M.J. van Wegberg, Bruno Lefort, François Labarthe, Francjan van Spronsen, François Maillot

**Affiliations:** 1 Université de Tours, INSERM, Imaging Brain & Neuropsychiatry iBraiN U1253, 37032, Tours, France; 2 Metabolic Consulting, Panorama Street 139, 72766 Reutlingen, Germany; 3 Department of Pediatrics, Endocrinology, Diabetology, Metabolic Diseases and Cardiology of the Developmental Age, Pomeranian Medical University in Szczecin, Szczecin, Poland; 4 Division of Metabolic Diseases, Beatrix Children’s Hospital, University Medical Center Groningen, Groningen, The Netherlands; 5 Department of Pediatrics, university hospital of Tours, Tours, France; 6 Department of Internal medicine, university hospital of Tours, Tours, France

**Keywords:** Phenylketonuria, Adults, Cardiovascular disease

## Abstract

Studies in adults with PKU have mainly focused on the neuropsychiatric complications that may arise in individuals who are unable to maintain the recommended lifetime diet. Some recent epidemiological studies suggest to consider other complications. As such, cardiovascular (CV) issues have been the subject of few studies to date. The aim of this review is to gather and discuss data from the literature on the traditional risks of CV complications in PKU, a potential CV phenotype in this population and the various non-traditional risks and potential associated mechanisms. The reported prevalence of comorbidities suggests an increased risk of CV complications in adults with PKU, mostly in late-diagnosed patients. Studies about a specific CV phenotype associated with PKU are suggestive, although further studies are needed. The data on oxidative stress in this population are consistent and confirm an increased CV risk. Regarding other potential mechanisms, it is not possible to conclude whether adult PKU patients have low grade inflammation, dyslipidemia, kidney impairment or if they have hyperhomocysteinemia. It would be of interest to measure potential biomarker associated with CV complications, such as homocysteine, asymmetric dimethylarginine and kynurenines (quinolic acid).

## Introduction

Phenylketonuria (PKU, OMIM 261600) is an inborn metabolic disease with an incidence of 1:24 000 living births in Europe [1]. PKU is mostly due to pathogenic variants of the phenylalanine hydroxylase (PAH) gene but hyperphenylalaninemia (HPA) may also be caused by mutations in tetrahydrobiopterin (BH4) metabolism [2] or in the DNAJC12 gene which encodes a co-chaperonne interacting with the PAH [3]. Mutations in PAH lead to a loss of function or even total inaction of the enzyme, depending on the locus [4]. PAH converts phenylalanine (Phe) to tyrosine (Tyr) and its dysfunction leads to an accumulation of both systemic and cerebral Phe associated with microcephaly, epilepsy, behavioral issues, and intellectual disability [5]. Over the past 60 years, newborn screening has made it possible to introduce a low Phe diet from an early age, allowing a normal physical and intellectual development of affected children [6]. According to current guidelines, such diet should be maintained for life [7, 8]. However, patients’ compliance decreases with age, particularly from adolescence to adulthood [9]. The effect of lifelong adherence to the low Phe diet, its cessation or discontinuation in adulthood is still poorly understood [10]. Classically, concerns about long term outcome in adults with PKU are focused on neurocognitive and psychiatric complications along with behavior issues [11] but some new questions about systemic manifestations of PKU occurring at the adult age have been recently raised [12]. Among some potential systemic features of PKU, cardiovascular (CV) complications have been questioned in the recent literature. As the incidence of cardiovascular disease increases significantly with age [13], the issue of CV damage is of importance for the management of an ageing population, considering that the first neonatally screened PKU patients are now approaching 60 years of age. Thus, the aims of the present review were to gather data from the literature about CV risk in PKU and potential specific CV phenotype of PKU as well as to discuss mechanisms that could be involved in such potential CV complications.

**Fig. 1:**
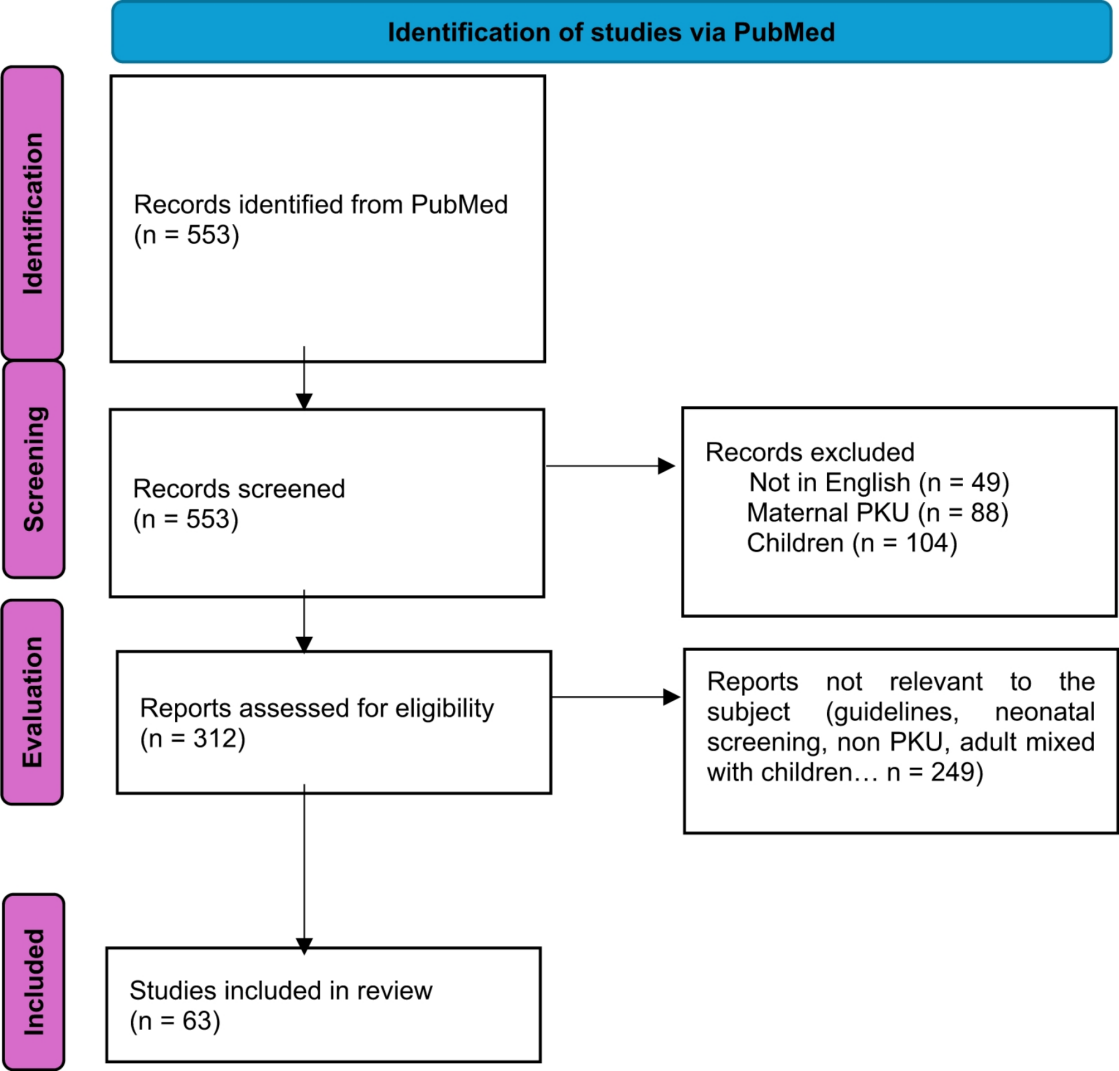
Flowchart showing article selection process. Based on title, articles not in English or dealing solely with children or the maternal syndrome of PKU were excluded. The remaining articles were then selected by reading the abstract or the article itself, when the abstract did not allow us to conclude whether the article was relevant to the subject in question. The remaining articles have been included in the review

We conducted a literature search on PubMed using the following entry: “phenylketonuria” AND (“heart” OR “cardiovascular” OR “atherosclerosis” OR “obesity” OR “overweight” OR “renal” OR “kidney” OR “inflammation” OR “cytokine” OR “lipids” OR “lipoprotein” OR “oxidative stress” OR “insurance claim” OR “microbiota” OR “homocysteine”). The first selection of articles was based on title reading. The remaining articles were selected by reading the abstract or the article when the abstract did not allow us to conclude on the relevance of the article to the subject. Only articles concerning adults (sometimes mixed with adolescents) were included in this review. No time interval was considered. Only articles written in English were included. The initial search identified 553 articles. 241 articles were then excluded (non-English articles, articles concerned solely with children or maternal PKU). 249 articles were then excluded according to their relevance to the subject. 63 articles have been selected for inclusion in this review. The process is summarized in flowchart Fig. 1. Finally, we took the option to present our results through 3 main questions: (1) is there an increased CV risk in adult patients with PKU? (2) is there a specific CV phenotype in PKU? (3) what could be the potential mechanisms involved in CV complications of PKU?

### Is there an increased CV risk in adult patients with PKU?

Classically, main risk factors for CV disease are, age, sex, obesity, smoking habit, sedentary lifestyle, essential hypertension, diabetes mellitus and dyslipidemia, as well as familial CV diseases [14]. Some ethnicities have a higher prevalence of certain CV risk factors such as diabetes mellitus, hypertension, dyslipidemia and obesity [15]. The definition of risk is associated with the probability of the occurrence of an undesirable event such as CV complications in this case [16]. Some algorithms calculate this probability based on a patient’s various risk factors, generally over a 10-year period [17]. Based on health insurance data contained in the research database of the institute for applied health research of Berlin “InGef”, Trefz et al. (2019) studied the prevalence of comorbidities in 377 German early-treated adults PKU and compared to matched controls (*n* = 3770) without distinction of diet and early or late diagnosis of PKU [18]. Most of the therapeutic agents prescribed were for essential hypertension. This comorbidity is the most frequent in PKU but is comparatively recurrent in the control population. The prevalence of comorbidities such as dyslipidemia, overweight/obesity, chronic ischemic heart disease and diabetes mellitus (type unspecified) was significantly higher in PKU patients as compared to healthy controls. Using a similar methodology, a study using three research databases from United States MarketScan ^®^, also reported a significant increase in overweight and essential hypertension prevalence in PKU patients (*n* = 3691) compared to matched controls [12]. Recently, a French study used health insurance claims data from the French SNDS (Système National des Données de Santé) database to report significantly increased prevalence of hypercholesterolemia, diabetes mellitus (type unspecified), ischemic heart diseases, essential hypertension and obesity in the general adult PKU population (*n* = 2175) compared to healthy matched controls (*n* = 10743) [19]. This study also showed no ischemic heart disease event in early-diagnosed adult PKU patients (prevalence 0%, *n* = 1528). Some therapeutics agents were significantly more prescribed to early treated adults with PKU, including pharmaceuticals for the CV system. With regard to overweight and obesity, a recent systematic review concluded that obesity and overweight are common in PKU patients, although the difference with the general population is debatable [20].

On the other hand, a recent study focused exclusively on the presence of comorbidities in a cohort of late-diagnosed PKU patients born before the introduction of newborn screening in 1972 in France (*n* = 647), compared to healthy subjects (*n* = 3228) [21]. These patients suffered significantly more from essential hypertension, chronic kidney disease, diabetes mellitus, hypercholesterolemia, obesity and ischemic heart disease. Most of these results were also reported by Trefz et al. (2019), except for obesity and essential hypertension [18]. Taken together, these results suggest an increased risk of cardiovascular complications potentially associated with hyperphenylalaninemia during the patients’ lifetime. Although early-treated patients were treated over a variable period depending on compliance and were younger than the patients recruited for this study, they appear to present a relatively comparable profile in terms of prevalence of traditional cardiovascular disease risks compared to healthy controls. This could indicate a potential deleterious role for hyperphenylalaninemia and, by analogy, non-compliance with the Low-Phe diet and its potential link with CV complications. It would be interesting to conduct a study comparing late-diagnosed patients with compliant and non-compliant early-treated patients with controls to confirm these hypotheses.

A recent report from a consortium of international experts on the management of PKU in adults identifies overweight, type 2 diabetes mellitus and chronic kidney disease as the comorbidities that require the most attention [22]. In summary, it would appear that the multiple comorbidities reported in adults with PKU point to the possibility that they have an increased risk for CV disease, and mostly in late treated patients.

### Is there a specific CV phenotype in PKU?

Studies that have directly investigated the CV features of PKU patients remain scarce. Of these, Tanacli et al. (2021) reported thinner cardiac walls, dilated left ventricle and reduced left ventricular ejection fraction in young adult PKU (*n* = 39); reflecting a potential early stage of dilated cardiomyopathy [23]. These changes seem to intensify as Phe increases. Most patients of this study were above current recommendations for blood Phe levels (mean Phe ± SD = 924 ± 330 µmol/L). In addition, it was shown that PKU patients (*n* = 41, mainly adolescents, different forms of PKU) have significant arterial stiffness compared to controls [24]. Interestingly, this study reported a positive correlation between blood Phe levels (83% of the patient had adequate median annual blood Phe) and arterial stiffness. Another study showed that resting heart rate, systolic and diastolic blood pressure were increased in PKU adults, supporting the data of Hermida-Ameijeiras et al. (2017), regarding arterial stiffness [25]. However, the data of Azabdaftari et al. (2019) did not find a correlation between CV impairment and concurrent blood Phe levels (mean Phe ± SD = 1172 ± 380 µmol/L) [25]. It is important to note that the PKU population studied (*n* = 23) had a significant increase in BMI compared to controls which may influence the nature and interpretation of these results [26]. In another study Htun et al. (2015) investigated whether there was an increased risk of atherosclerosis in adult early-treated PKU patients (mean phe ± SD = 938 ± 36 µmol/L) [27]. They reported no difference in arterial stiffness and other markers of early atherosclerosis changes. These differences may be due to the different techniques of investigations used between studies.

Interestingly, recent non PKU studies have shown that altered Phe catabolism plays an important role in heart ageing and is even used as a diagnostic tool [28–31]. This would be due to an age-dependent decline in hepatic PAH activity as demonstrated in an animal model study [32]. In the same study, the authors also showed that injection of Phe (200 mg/kg) to young wild type mice partly mimicked the cardiac phenotype of old mice. The cardiac phenotype of old mice was largely improved when the mice were treated with BH4 or with a low-Phe diet. It is then possible to assume that this phenomenon of senescence of the cardiac tissue is accelerated in PKU patients, especially in case of poor metabolic control.

### What could be the potential mechanisms involved in CV complications of PKU?

#### Inflammation

Inflammation is increasingly recognized as being involved in the initiation and progression of atherosclerosis and CV disease [33] but it remains poorly studied in PKU patients. High sensitivity CRP is a good indicator of inflammation, although it is not specific and may not be associated with CV complications [34]. Azabdaftari et al. (2019) reported a significant increase in CRP and serum amyloid A (SAA) protein (for review [35]) in PKU patients. Again, in this study, it is important to highlight the significantly higher BMI compared to controls. Being overweight is known to be the cause of a particular inflammatory context [36]. A few studies have looked at plasma cytokine concentrations to detect potential systemic low-grade inflammation in PKU. Deon et al. (2015), reported a significant increase in IL-1β, IL6 in PKU patients in late adolescence (*n* = 10, mean ± SD = 16.6 ± 1.27 years old) [37]. However, the small number of patients, the lack of indication of BMI in patient and controls, and the fact that these patients have been lately diagnosed with PKU, imply great caution in the interpretation of these data. Interestingly, Faverzani et al. (2023) showed increased levels of IL-1β, IL2, IL6, IL8 and TNFa and anti-inflammatory cytokine IL4 in late-diagnosed adolescent patients compared to healthy controls [38]. This could explain the results found by Deon et al. (2015), as these two studies indicate that high exposure to Phe during childhood could be a source of inflammation [37].

In contrast, Mozrzymas et al. (2016) also measured these parameters in young early-treated adult PKU patients on diet (*n* = 20, mean ± SD = 25,25 ± 4,55 years) [39]. They reported no significant difference between patients and controls for plasma IL6 and IL8 indicating that there would be no systemic inflammation in adult PKU patients on diet. Recently, Giret et al. (2023) studied a cohort of early-treated PKU adult (*n* = 20, mean ± SD = 36,3 ± 8,5 years) with poor metabolic control (mean Phe ± SD = 1179 ± 550 µmol/L) compared to healthy matched control (*n* = 20) for several pro-inflammatory cytokines and CRP [40]. No significant difference was reported between the two groups. Stroup et al. (2017), studied a large panel of cytokines between PKU patients (*n* = 27) on amino-acids mixture (AA-MF) and a control population (*n* = 254) [41]. They found a significant increase in pro-inflammatory cytokines such as TNFa, IFNg, IL-1β, IL6, IL12, IL17, and anti-inflammatory IL10. This study included patients from a previous clinical trial with various forms of PKU, more than a third of whom being responsive to sapropterin; this involving a wide range of phenylalaninemia (201 to 1418 µmol/L) [42]. The high heterogeneity of this cohort implies great caution in the interpretation of such data. These conflicting results do not allow us to conclude clearly about the existence of any inflammation in adult PKU patients.

Interestingly, a study that looked at kynurenines in PKU reported changes in this metabolic pathway [43]. Briefly, tryptophan (Trp) is mainly used for the secretion of kynurenines which ultimately allow the regeneration of NAD+, essential for energy metabolism [44]. This pathway is particularly solicited in an inflammatory context [45]. A greater proportion of Trp is said to be metabolized via this pathway when AA-MF is consumed [46], which could be linked to the inflammatory context reported by Stroup et al. (2017) when AA-MF is consumed [41]. It is not possible today to make a statement in view of the conflicting results regarding inflammation in PKU as stated above. An in-depth study of kynurenines in adult PKU patients could be of interest in the study of inflammation. It is also important to note that the accumulation of some of the metabolites of this pathway is strongly suspected to have an impact in the development of CV diseases [47].

#### Gut microbiota

Gut microbiota is largely shaped by our eating habits [48]. Most PKU patients are treated with a low-Phe diet. High protein containing foods such as meat, fish, eggs, cheese, nuts, seeds, soya and flour-based foods are completely avoided in this context. Besides fruits and vegetables, special manufactured low protein food is incorporated in their diet. Moreover, PKU patients take an amino acid supplement that contains all essential amino acids except Phe.

This Low-Phe diet has particular characteristics and is highly likely to involve changes in microbiota [49]. Alterations in the composition of the gut microbiota, or even dysbiosis, can lead to a number of changes in digestion, metabolism, gut barrier permeability and immune responses [50]. Microbiota can also exert pro-atherosclerotic effects through the alterations of the productions of several metabolites, being implied in essential hypertension through an effect on blood pressure and is suspected of being involved in the progression of heart failure (for review [51]).

Most of the studies focusing on the microbiota in PKU concern children [49, 52–55]. Mancilla et al. (2021), reported a change in the gut microbiota of adults with PKU compared to controls. The genus most impacted would be faecalibacterium sp [56]. These bacteria are beneficial through the production of butyrate and their anti-inflammatory action [57, 58]. Timmer et al. (2021), also reported a change in the gut microbiota compared to controls, notably a decrease in the presence of the phylum Ruminococcacae (of which faecalibacterium is a part), as well as an increase in Lachnospiracae [59]. McWorther et al. (2022) studied the microbiota of a small sample of adults with PKU (*n* = 6) on diet and pegvaliase [60], but there was no control sample to include these results in this review. In sum, the change in diversity, butyrate-producing and anti-inflammatory bacteria suggests a pro-inflammatory dysbiosis. This remains a hypothesis as, for example one study reported the absence of digestive system inflammation in PKU [61]. Studies involving larger samples with a metabolic and nutritional history are needed to confirm such hypothesis.

One of the major alternatives to synthetic AA-MF is the use of glycomacropeptide (GMP-MF) which is a low-Phe peptide derived from cheese manufacture [62]. This peptide have several properties of interest, including a prebiotic and anti-inflammatory effect [63]. An animal model study reported a significant decrease in IL-1β, IFNg and CM-CSF cytokines in animals consuming GMP-MF compared to the group receiving AA-MF [64]. Sawin et al., (2015), observed no difference in the plasma cytokine profile in the same comparisons [65]. Pinto et al. (2017) found no differences regarding CRP in the same comparison [66]. It would be necessary to repeat these experiments on a large cohort of adult PKU patients to confirm these results.

#### Methionine and arginine metabolism

Hyperhomocysteinemia has been recognized for many years as a factor that can promote the development of CV disorders [67]. Homocysteine (Hcy) is thought to be involved in endothelial dysfunction, smooth muscle cell proliferation and impacts on the elastic properties of arteries [68]. Briefly, Hcy is part of the folate cycle and is a metabolite of methionine (Met), its degradation leads to the synthesis of cysteine [69]. B-vitamins (B2, B6, B12) are essential for the proper functioning of this metabolism and prevent Hcy accumulation [70]. The intake of these vitamins in PKU may be problematic due to patients’ poor access to natural protein sources [71], especially vitamin B12 which is mainly found in animal products [72]. AA-MF are supplemented with vitamins and micronutrients that allow compliant adult patients to be within reference standards for most of these micronutrients [73, 74]. A recent meta-analysis did not report deficiency of any of these vitamins in the blood of PKU patients consuming AA-MF [75]. Intriguingly, it would be the non-compliant patients who would be prone to deficiencies, probably due to the lack of use of AA-MF and/or a poor-quality diet [76, 77]. In sum, there should be no defect in the metabolic pathway described above, which should result in moderate homocysteinemia.

Hcy has been measured in PKU children and adolescents with highly conflicting results [71, 78–83]. This is probably due to the different measurement techniques used and the heterogeneity of the cohorts in terms of age and diet. Indeed, although some studies distinguish between good and poor control, there is no information about the diet formula followed by patients in many of them. To our knowledge, only two studies have measured homocysteine in a cohort of exclusively adult PKU patients. Hvas and Nielsen (2006) found that, on average, patients were within reference standards, but hyperhomocysteinemia was detected in 29% of patients studied. These patients had been on an unrestricted diet since adolescence. Lucock et al. (2002) found no difference compared with a control population when studying a cohort of PKU patients with the same characteristics as Hvas and Nielsen (2006). Studies involving a larger sample that would include patients with various metabolic controls are needed to further investigate the issue.

It is also important to note that Met metabolism is strongly linked to arginine (Arg) metabolism [84]. Arg is involved in nitric oxide (NO) synthesis. Met serves as a methyl group donor to enable the synthesis of methylated Arg derivatives, such as symmetrical or asymmetrical dimethyl Arg (ADMA). ADMA is an endogenous inhibitor of nitric oxide synthase (NOS). A significant decrease in ADMA in PKU children and adolescents on Low-Phe diet has been reported [81, 85]. These results are in agreement with other studies including children and adults with different forms of PKU without group distinction [86, 87]. This decrease could induce a dysfunction of NOS inhibition, which is known to be a major player in CV homeostasis [88]. This probable excess of NOS activity can lead to changes in cardiomyocyte function and even heart failure [89]. This excess can also induce dysfunction of the vascular endothelium which can lead to the development of atherosclerosis [90].

#### Mitochondrial dysfunction and oxidative stress

The particular diet that PKU patients follow may cause oxidative stress through limited antioxidant intake or non-compliance with the diet leading to HPA. Oxidative stress is considered to be an imbalance between endogenous antioxidant mechanisms and the production of reactive oxygen species (ROS) [91] and is thought to be strongly implicated in CV disease risk [92]. Phe and its metabolites themselves are thought to impact mitochondrial function and free radical production due to a strong correlation of Phe and oxidative stress in adult PKU patients [93]. HPA is also thought to downregulate many genes involved in antioxidant mechanisms, as demonstrated by the analysis of leukocytes from adult PKU patients [94]. A Phe catabolite which is over-represented in PKU, phenylpyruvate, would also inhibit the enzyme glucose 6 phosphate dehydrogenase which is essential for the production of pyruvate and NADPH, these two compounds being also essential for the proper functioning of energy and antioxidant metabolism [95]. Dobrowolski et al. (2022) have identified strong deregulation of energy metabolism accompanied by oxidative stress in the brain. Indeed, they observed a decrease in the NADH/NAD ratio, suggesting a defect in the first subunit of the mitochondrial respiratory complex; this seems to be confirmed by a respirometry study [96]. Like neurons, cardiomyocytes are cells with high energy demands, making them the most oxygen-demanding organ in the body [97]. It is thus possible to assume that mitochondrial dysfunctions are detectable at the cardiac level of PKU adults. Dysfunctions in the respiratory chain of these organelles at the level of cardiomyocytes may play a crucial role in cardiac pathogenesis via the induction of a decrease in the ATP pool, oxidative stress and cellular damage that may ultimately lead to apoptosis [98]. Mitochondrial dysfunction is a well-known comorbidity of PKU [99], but has not been studied in the heart, such studies are needed to confirm these hypotheses.

HPA is also thought to be a source of DNA damage through the generation of ROS [83, 100] and could be decreased by the addition of antioxidants such as L-carnitine [101]. Another in vitro study shows that the addition of L-carnitine prevents oxidative stress induced by Phe and its metabolites [102]. L-carnitine is mainly derived from red meat [103] and is reportedly deficient in PKU patients with good metabolic control [104]. L-carnitine supplementation could partially correct oxidative stress in PKU patients [105]. PKU patients are also at risk of deficiencies in ubiquinone 10 which is involved in the mitochondrial respiratory chain [106–109]. All of these studies point to significant oxidative stress that is directly related to Phe levels in PKU patients. However, the diet itself could be the cause of antioxidant deficiency as illustrated by L-carnitine deficiencies [104]. Adequate antioxidant supplementation should result in a decrease in oxidative stress in compliant patients. Non-compliant patients without nutritional intervention would remain heavily impacted, which could be a source of CV complications.

#### Dyslipidaemia

Dyslipidaemia is a major factor in the development of CV complications [110]. The Low-Phe diet is likely to induce dyslipidaemia due to a near-vegetarian diet. The lipoprotein profile of PKU children has been extensively studied [79, 111–119], but remains understudied in adults. Furthermore, this profile changes with age and therefore does not reflect the profile of adults [120]. This limits the interpretation of studies that have included children and adults without distinction [121–123]. Azabdaftari et al. (2019) reported a different lipoprotein profile in PKU adults compared to controls that would be associated with dyslipidaemia [25]. Once again, it is important to note the difference in BMI between the two groups studied. Tanacli et al. (2021) reported a similar lipoprotein profile compared to Azabdaftari et al. (2019) when comparing patients with a phenylalaninemia of more than 1200 µmol/L to patients with a phenylalaninemia of less than 900 µmol/L [23]. In the same way, Fernandez-Crespo et al. (2023) [124] and Htun et al. (2015) [27] showed a decrease in HDLc and an increase in triglycerides compared to controls. Two studies disagree with the last ones, reporting an increase in the LDLc/HDLc ratio. Williams et al. (2015) reported a significant reduction in LDLc compared with the control population [125]. It is important to note that 72% of the study population was obese (mean BMI ± SD = 30.3 ± 1.8 kg/m2); this may explain the differences with the previously cited studies. These results are also reported by Cannet et al. (2020) using a nuclear magnetic reasoning approach [126]. The BMIs are comparable to Azabdaftari et al. (2019), although there is no specification on the significance of the difference between control and patients. Another factor that may explain these differences is the variety of protein substitutes used by patients. Indeed, some formulas are supplemented with long-chain fatty acids and fats [127], which may influence the lipoprotein profile. It would be necessary to repeat studies on this subject, attempting to have uniform cohorts in terms of diet and BMI. The above studies are summarized in Table 1. Interestingly, one study reported a reduction in LDLc after six months of treatment with GMP [128], which could represent cardioprotective properties [129].

**Table 1:** Summary of lipoprotein profile studies in adult PKU patients

Lipoprotein profile studies	Age: patient vs. control (if present)	BMI: patient vs. control (kg/m2)	Phe (µmol/L)	Findings
**Fernando-Crespo et al. (2023)**	36 ± 9 vs. 39 ± 10	26 ± 6 vs. 24 ± 4	550 ± 248	Decrease of TC, HDLc, LDLc, increase of TG
**Tanacli et al. (2021)**	30.5 ± 8.7	25.7 ± 5.0	924 ± 330	• increase of TC in patient with Phe ≤ 1200 µmol/L vs. patient with Phe ≤ 900 µmol/L, • increase of TC in patient with Phe ≤ 1200 µmol/L vs. patient with 900 < Phe ≤ 1200 µmol/L • increase of LDLc/HDLc in patient with Phe ≤ 1200 µmol/L compared to patient with Phe ≤ 900 µmol/L
**Azabdaftari et al. (2019)**	30.8 ± 8.4 vs. 30.1 ± 9.1	27.6 ± 5.4 vs. 23.4 ± 6.4 *	1172 ± 380	increase of TC, HDLc, LDLc/HDLc, non HDLc
**Htun et al. (2015)**	28.1 ± 0.96 vs. 26.2 ± 0.5	24.3 ± 0.7 vs. 21.9 ± 0.3	940 ± 40	Increase of TC and decrease of HDLc compared to control
**Williams et al. (2015)**	31 ± 12	30.3 ±1.8 ; 72% were obese, 14% overweight, 14% normal BMI	1194 ± 522	LDLc levels were lower than in the age-matched community population (male and female)
**Cannet et al. (2020) ²**	38.7 (range 30–54) vs. 35.2 (range 30–45)	27.2 (range 20.7–51.3) vs. mean of 23.9 (range 21.3–29.8)	899 (range 50–1318)	Decrease of total cholesterol and LDLc (decrease in 22 LDLc subclasses)

² = standard deviation not provided, * = significant, abbreviations: TC = total cholesterol, TG, triglycerides

Altered plasma lipid and erythrocyte profiles have also been extensively studied in PKU children or mixed with adults [130–133] and remain poorly studied in adults. Stroup et al. (2018), reported numerous changes in the membrane constitution of PKU adult erythrocytes [134]. These results are in agreement with Moseley et al. (2002) [135]. On the other hand, Htun et al. (2015) reported no difference in the lipid profile of erythrocytes [27]. In sum, studies concerning potential dyslipidemia in PKU adults are somewhat contradictory, probably due to differences in cohort and dietary follow-up. Further studies are needed to confirm this in order to conclude on potential dyslipidemia and associated CV risks.

#### Kidney function

In other part, renal impairment or chronic kidney disease implies an increased risk of developing heart disease [136]. Among these, essential hypertension is known to have a significant correlation with the development of CV disorders [137] and is among the traditional risks of developing CV complications. A few studies have looked at renal function in PKU adults. Among them, Hennermann et al. (2013) reported essential hypertension, hypercalciuria, proteinuria and finally a reduced glomerular filtration rate in young PKU adults fed AA-MF since childhood [138]. Glomerular filtration rate decreased significantly with increasing AA and total protein intake. In fact, AA-MF induces a rapid increase in plasma AA, reaching a peak before decreasing rapidly [139], leading to an increase in renal workload. A persistent increase in renal workload can induce hyperfiltration, damaging nephrons and resulting in a reduced filtration rate [140]. Thus, long-term AA-MF consumption could be a major factor in the onset and progression of chronic kidney disease in PKU patients [141]. A recent study following adult PKU patients over 10 years according to metabolic control also investigated renal function [142]. Patients with good adherence to the AA-MF-supplemented low-Phe diet had better renal parameters than those with suboptimal control. They reported a moderate negative correlation between glomerular filtration rate and Phe. This study suggests that prolonged hyperphenylalaninemia would have a negative impact on renal function. A recent study of PKU adults on AA-MF containing phenylalaninemia above recommended levels on average (mean Phe ± SD = 778.55 ± 218.94 µmol/L) suggests that they are within normal limits for all measured renal function parameters [143]. Despite the significant decline in glomerular filtration rate after 10 years, as well as in the group with poor control compared with good metabolic control, the patients studied by Prepok et al. (2023) are within reference values [142]. The same observation applies to Hennerman et al. (2013), who found several defective parameters in the renal function of a fraction of the patients studied [138].

A study on PKU murine models reported an equivalent increase in renal mass when consuming AA-MF or a high-protein diet (high Phe casein diet) compared to GMP-MF consumption [64], indicating a potential beneficial effect of GMP-MF on renal function by reducing renal workload. These results concerning renal workload were also reported by Stroup et al. (2017) [144]. The comparison between AA-MF and GMP-MF on renal function was also conducted mainly on PKU adults (*n* = 30, 5 adolescents of 15–17 years) consuming one or the other three weeks before the exams [46]. They reported no difference between the two groups for the various renal parameters measured. GMP-MF administration may have been too short, with Solverson et al. (2012) administering GMP-MF for almost 20 weeks to the mice. Further study is needed to conclude on the subject [64].

In conclusion, it would appear that AA-MF intake has a negative impact on renal function. However, the impact of high phenylalaninemia would be even more deleterious. It is important to note that despite the kidney function decline, the patients remain within the reference values. The potential beneficial impact of GMP-MF on renal function needs to be further investigated. A fairly recent survey of numerous European reference centers have revealed that renal function is poorly monitored [145] in this population, which seems at risk of developing renal complications and by analogy increasing the risk of CV complications.

## Conclusion

**Fig. 2:**
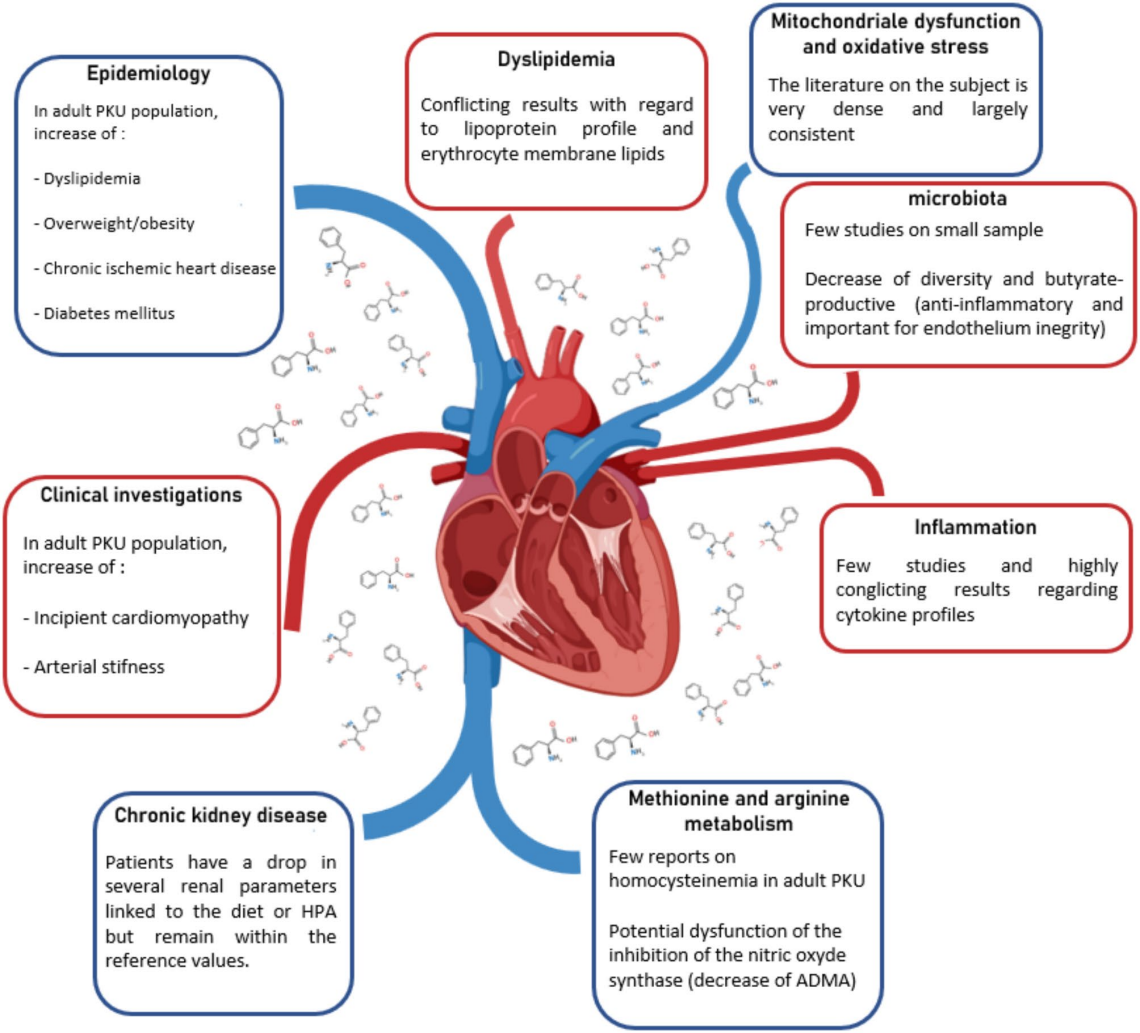
Summary of results concerning traditional CVD risk and potential associated mechanisms. Heart image comes from BioRender.com

The overall results discussed in this narrative review suggest an increased risk of CV complications in the adult PKU population. The findings regarding oxidative stress in this population seem to be generally consistent and reflect a potential risk profile. Further studies of inflammation in PKU are needed to determine whether there is any systemic inflammation that may promote the development of CV complications. The same remark applies to potential dyslipidaemia and kidney impairment. A summary is shown in Fig. 2. It would also be interesting to measure some biomarkers associated with CV risk such as asymmetric dimethylarginine, homocysteine and kynurenines (quinolinic acid). However, it is currently not possible to confirm a potential increase in CV complications in adult PKU patients. The present review highlights the limited amount of information about adults with PKU. Further studies in such group of patients are needed to assess CV complications in this ageing population and improve follow-up as well as CV disease prevention.

## Data Availability

There is no data sharing as no dataset has been generated or analyzed for this article.
